# Patterns of Chemical Diversity in the Mediterranean Sponge *Spongia lamella*

**DOI:** 10.1371/journal.pone.0020844

**Published:** 2011-06-17

**Authors:** Charlotte Noyer, Olivier P. Thomas, Mikel A. Becerro

**Affiliations:** 1 Center for Advanced Studies of Blanes (CEAB, CSIC), Blanes, Spain; 2 Université de Nice - Sophia Antipolis, Laboratoire de Chimie des Molécules Bioactives et des Arômes, LCMBA-UMR 6001 CNRS, Nice, France; National Institute of Water & Atmospheric Research, New Zealand

## Abstract

The intra-specific diversity in secondary metabolites can provide crucial information for understanding species ecology and evolution but has received limited attention in marine chemical ecology. The complex nature of diversity is partially responsible for the lack of studies, which often target a narrow number of major compounds. Here, we investigated the intra-specific chemical diversity of the Mediterranean sponge *Spongia lamella*. The chemical profiles of seven populations spreading over 1200 km in the Western Mediterranean were obtained by a straightforward SPE-HPLC-DAD-ELSD process whereas the identity of compounds was assessed by comparison between HPLC-MS spectra and literature data. Chemical diversity calculated by richness and Shannon indexes differed significantly between sponge populations but not at a larger regional scale. We used factor analysis, analysis of variance, and regression analysis to examine the chemical variability of this sponge at local and regional scales, to establish general patterns of variation in chemical diversity. The abundance of some metabolites varied significantly between sponge populations. Despite these significant differences between populations, we found a clear pattern of increasing chemical dissimilarity with increasing geographic distance. Additional large spatial scale studies on the chemical diversity of marine organisms will validate the universality or exclusivity of this pattern.

## Introduction

The current global loss of species has surpassed scientific interest, placing biodiversity conservation in political agendas and media around the globe [1], [2]. But diversity is a complex and dynamic concept that applies below and beyond the species level [3]. Specifically, the intra-specific level of diversity is particularly relevant for species evolution [4]. It occurs at the within and between population level and provides the variance on which natural selection acts [4], [5]. Many organisms produce secondary metabolites that play multiple functions in nature [6]. Secondary metabolites have evolved under the pressure of natural selection and represent ecological responses of organisms to their environment [5], [6], [7]. Intra-specific diversity in secondary metabolites is therefore crucial to understand species biology, ecology, and evolution, yet, it has received partial attention. This is particularly true in marine environments [5], [8].

Marine benthic organisms provide unique opportunities to investigate chemical diversity. Sessile or slow moving organisms without effective escape mechanisms or structural protection are likely to be chemically defended [9]. Sessile organisms also are under intense pressures to acquire and maintain space on where to live [10], to gain protection from ultraviolet radiation [11] or to prevent from fouling [12]. Intra-specific information on their chemical diversity is fundamental to look for general patterns of secondary metabolite production and to understand the processes that produce chemical diversity in benthic communities. Available evidence suggests that intra-specific spatial and temporal variation in secondary metabolism is widely distributed in the benthic realm including algae [13], [14], [15], sponges [16], [17], [18], cnidarians [19], tunicates and bryozoans [19], and worms [20].

Sponges are major components of benthic communities and among the richest source of secondary metabolites isolated from marine organisms [21], [22], [23]. Unsurprisingly, chemical ecology has contributed significantly to advance in our understanding of sponge ecology [24]. Many sponge secondary metabolites are known to deter feeding, confer competitive advantages, and protect against fouling among other ecological roles [8], [25]. But the demand for studies on sponge chemical ecology looms large [24] and the study of chemical diversity at a geographic scale is an area that has received little attention. The sponge *Aplysina aerophoba* shows significant differences in the concentration of bromotyrosine derivatives in multiple locations around the Canary Islands but the same secondary metabolites were present in all specimens [26]. In a distinct way the sponge *Acanthella cavernosa* shows remarkable variation in the sesquiterpene composition of specimens from multiple locations [27]. Variation at a geographic scale is fundamental to understand population genetics, demographic history, and evolution of benthic species [28], [29]. Similarly, geographic patterns of chemical variation can be critical to understanding the evolution and function of secondary metabolites, as well as unraveling large scale patterns and a hidden chemical diversity that are poorly understood [8].

The secondary metabolites of the genus *Spongia* are well described [30], [31], [32], [33]. The sponge *Spongia lamella* is an Atlanto-Mediterranean species well studied for its chemical diversity and a careful examination of the secondary metabolites composition has already been established [31], [33]. Traditionally collected as a bath sponge [34], recent disease epidemics have decimated populations of *S. lamella* [34], [35], [36] and it is now considered an endangered species under Annex 3 of Bern and Barcelona Conventions [37]. Microsatellite markers previously described for this species revealed that populations were genetically differentiated and most of them must have suffered from recent demographic reductions [38], [39]. Loss of genetic diversity has a major impact on population survival especially for threatened species. It plays a crucial role in increasing extinction risk, and thus in depleting biodiversity [40]. Information on the chemical variability of this species is lacking especially at a geographic level. Loss of intraspecific variation in the production of secondary metabolites can also jeopardize species survival and limit the potential for direct and indirect uses of diversity for the future, such as identification of compounds of pharmaceutical or biotechnological interests [2], [41]. Given the potential applications of sponge secondary metabolites as new drugs or antifoulants [42], [43], [44], understanding the hidden chemical diversity of endangered species is a priority that could have strong conservation consequences.

In this paper we investigate the chemical diversity of *Spongia lamella* in seven Mediterranean populations distributed over 1200 km of coast. Diversity is a multifaceted concept based on i) the number of entities (richness) and ii) their abundances (evenness), iii) dissimilarities, iv) and functional roles [45]. Our study aimed at three out of the four pillars that support diversity as we quantified chemical diversity (richness/abundance), and dissimilarity of *S. lamella* at an intra-specific level and at two geographic scales (populations and biogeographic areas).

## Results

### Compound Identification and Quantification

**Figure 1:**
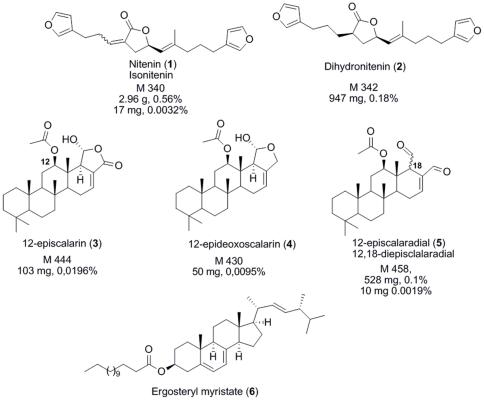
Secondary metabolites identified from *Spongia lamella*. Molecular mass and percentage in the extract (w/w) are included as described by Rueda and coworkers [33].

The HPLC-ELSD calibration curve of Nitenin (**1**), we obtained by HPLC purification, showed good linearity within the range of concentrations investigated (R^2^ = 0.999). The concentration of **1** in our samples was very close to that measured by the group of Salva (mean±se, 18.50±6.15 mg.g^−1^, dry mass) and varied between 6.30 mg.g^−1^ in Ceuta and 26.25 mg.g^−1^ in Els Bullents [33]. Other major compounds observed by our standardized HPLC-DAD-ELSD protocol were identified by comparison of MS and NMR data with literature but also on the basis of the metabolic composition described by the group of Salva [33] (Figure 1).

**Figure 2:**
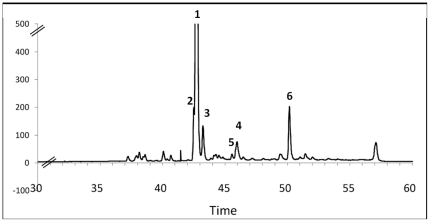
ELSD-HPLC chromatogram of *Spongia lamella* secondary metabolites. SPE-ELSD-HPLC chromatograms from desalting crude extract of *Spongia lamella*. The major compound identified as nitenin (1) was used as internal reference. Numbers are compounds described on Figure 2. (6) Second major compound described as an ergosterol ester.

The less retained compounds between 35 and 40 min were only visible at 210 nm in the UV spectrum. According to their retention times and mass spectra we assumed they could be assigned to the sterol derivatives previously described in *S. lamella* [31]. Dihydronitenin (**2**) and nitenin (**1**) were easily identified at RT 42.5 and 43 min after after HPLC purification and interpretation of NMR and MS analyses (Figures 1 and 2). The MS spectrum of the compound at RT 43.5 min exhibited *m/z* at 911.8 [2M+Na]^+^, 467.5 [M+Na]^+^, 445.7 [M+H]^+^, 385.5 [M-CH_3_CO_2_H+H]^+^ which strongly suggested the presence of 12-episcalarin (**3**) [46] also isolated as one of the major metabolites of this species [33]. Similarly, the MS spectrum of the compound at RT 46.0 min exhibited *m/z* 883.8 [2M+Na]^+^, 453.5 [M+Na]^+^, 353.4 [M-CH_3_CO_2_H-H_2_O+H]^+^ which suggested the presence of 12-epideoxoscalarin (**4**) [46]. Very close to this compound at RT 46.5 min the peak with *m/z* 879.8 [2M+Na]^+^, 451.5 [M+Na]^+^, 369.5 [M-CH_3_CO_2_H+H]^+^ evidenced the absence of an hydroxyl group and then suggested the presence of 12-episcalaradial (**5**) (Figures 1 and 2). The presence of these biogenetically related metabolites was also interpreted as a confirmation of their structure. The major apolar compound did not show any peak in the mass spectrum and showed very distinct UV profile as it strongly absorbed at 254 and 280 nm. After HPLC purification the structure of this compound was elucidated on the basis of NMR spectra interpretation and comparison with literature data. The compound at RT 50.5 min exhibited signals at *δ*_H_ 1.02 (d, *J* = 6.5 Hz, H-21), 0.94 (s, H-19), 0.92 (d, *J* = 6.7 Hz, H-28), 0.86 (H-26), 0.83 (H-27) ppm in its ^1^H NMR spectrum which were characteristic of an ergostane steroid. Furthermore, three unsaturations were evidenced in the steroid core by the signals at *δ*_H_ 5.57 (H-6), 5.38 (H-7), 5.20 (H-23) and 5.18 (H-22) ppm which were consistent with an ergosterol derivative. The signals at *δ*_H_ 2.35 (t, *J* = 7.1 Hz, 2H, H-2′) and *δ*_C_ 33.7 ppm indicated the presence of a fatty acid ester at O-3. The length of the alkyl chain was finally determined by integration of the signal at 1.29 ppm. Comparing with the literature data this compound was identified as the previously described ergosteryl myristate [47].

### Intra-specific Chemical Diversity

**Figure 3:**
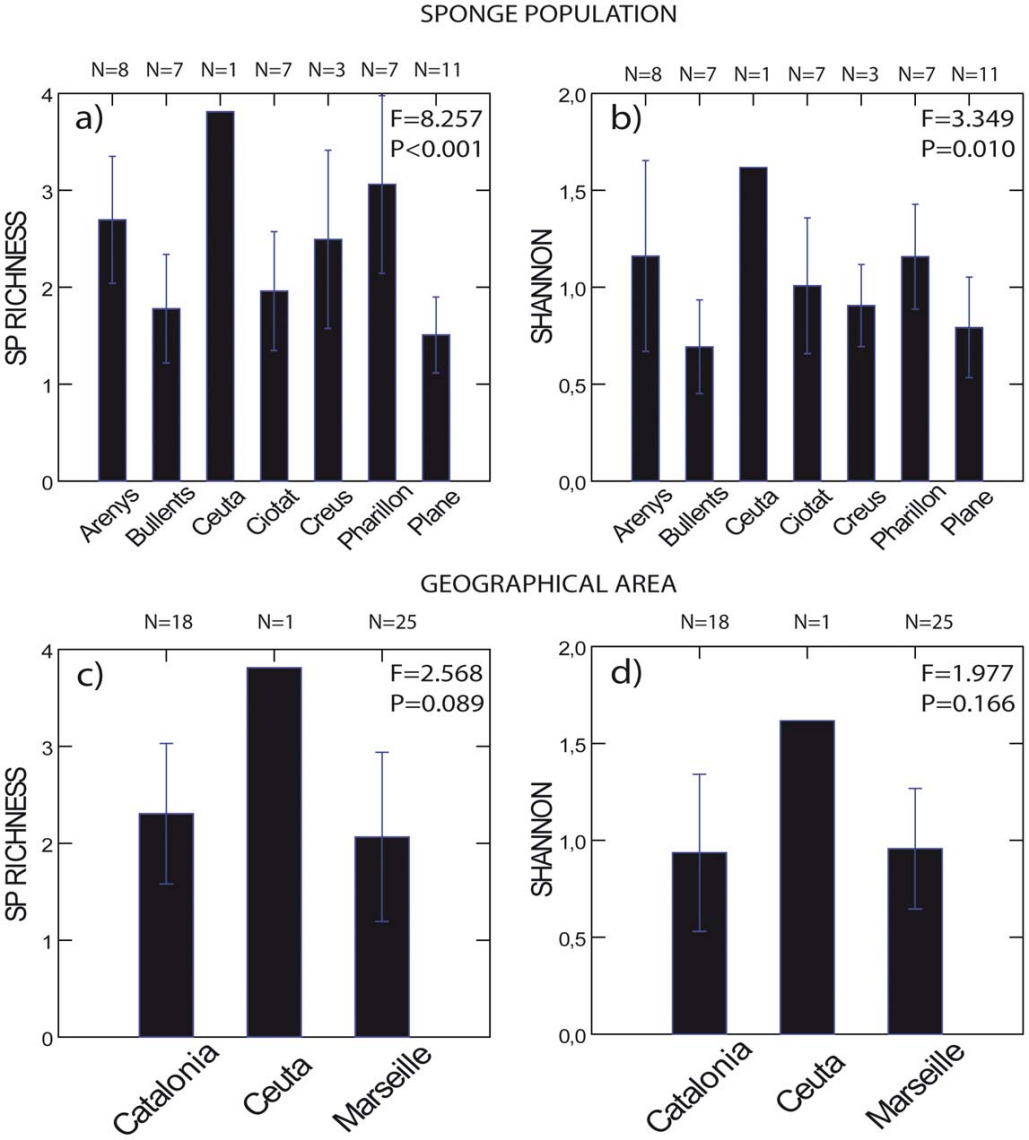
Chemical diversity of *Spongia lamella*. Chemical diversity was estimated in *Spongia lamella* at the population level a) and b), and at regional level c) and d) for peak richness and Shannon indices, respectively. Vertical bar is 1 standard deviation. Note lack of variation in Ceuta. A single specimen from this location was observed, sampled, and used in the analyses. One-way ANOVA: df = 6 at the population level, df = 2 at the regional level; F and P values included. See Table 1 for *Post hoc* Fisher's LSD tests.

At the population level, the average number of compounds ranged from six in Plane to eleven in Ceuta and Pharillon. Compound richness was low and varied from 1.51 for Plane to over 3 for Pharillon and Ceuta. Shannon index varied from 0.69 in Els Bullents to 1.61 in Ceuta. The sponge populations of Els Bullents and Plane showed lower compound richness (*F* = 8.257, P<0.001, Figure 3a, Table 1) and Shannon Index (*F* = 3.349, P = 0.010, Figure 3b, Table 1) than Ceuta, Pharillon, and Arenys. At the regional level, we found no significant differences in compound richness (*F* = 2.568, P = 0.089, Figure 3c) and Shannon index (*F* = 1.977, P = 0.166, Figure 3d).

**Table 1:** *Post hoc* Fisher's Least-Significant-Differences tests for *Spongia lamella* chemical diversity.

	Populations	SHANNON						
		Cio	Pla	Far	Cre	Bul	Are	Ceu
Species richness	Cio	-	NS	NS	NS	NS	NS	NS
	Pla	NS	-	**0.018**	NS	NS	**0.013**	**0.013**
	Far	**0.001**	**<0.001**	-	NS	**0.007**	NS	NS
	Cre	NS	**0.014**	NS	-	NS	NS	0.005
	Bul	NS	NS	**<0.001**	NS	-	**0.005**	**0.007**
	Are	**0.016**	**<0.001**	NS	NS	**0.004**	-	NS
	Ceu	**0.005**	**0.001**	NS	NS	**0.002**	NS	-

Species richness: below the diagonal; Shannon index: above the diagonal. Only values ≤5% are represented. NS: Non Significant.

Factor analysis (FA) of the chemical data led to six independent chemical factors that explained more than 84% of the total variance (Table 2). Both at local and regional scale, seasonal sampling did not influence chemical factors dissimilarities between populations and our data show the same degree of dissimilarity within and between seasons. In Marseille, winter chemical dissimilarities (10.45±1.29) were similar to winter-summer dissimilarities (13.01±2.07, *t*-*test*, P = 0.158). In Catalonia, the summer dissimilarity (16.20) fell within winter-summer dissimilarities (15.84±1.05, *t-test*, P = 0.717).

**Table 2:** Independent chemical factors (in columns) obtained after the Factor Analysis of chemical data.

Peaks	Chemical Factors					
	FC1	FC2	FC3	FC4	FC5	FC6
**18**	0.965	-	-	-	-	-
**19**	0.965	-	-	-	-	-
**17**	0.964	-	-	-	-	-
**23**	0.881	-	-	-	-	-
**20**	0.729	-	-	-	-	-
**21**	0.659	-	-	-	-0.691	-
**14**	0.653	-	-	-	-	-
**(5)**	-	-	-	-	-	0.746
**(6)**	-	0.916	-	-	-	-
**25**	-	0.913	-	-	-	-
**24**	-	0.877	-	-	-	-
**(4)**	-	0.719	-	-	-	-
**(1)**	-	-	-0.609	-	-	-
**5**	-	-	-0.852	-	-	-
**4**	-	-	-0.837	-	-	-
**9**	-	-	-0.808	-	-	-
**7**	-	-	-0.774	-	-	-
**2**	-	-	-	-0.891	-	-
**3**	-	-	-	-0.739	-	-
**8**	-	-	-	-0.638	-	-
**6**	-	-	-	-	-0.917	-
**(3)**	-	-	-	-	-	0.922
**12**	-	-	-	-	-	0.913
**% of total variance explained**	22.37	17.97	14.12	10.43	7.70	11.55

For a better interpretation, only peaks with absolute value greater than 0.600 are shown. Numbers in bracket are compounds from Figures 2 and 3.

Multivariate analyses of variance showed significant chemical differences between sponge populations (*Wilks' Lambda F* = 4.168, P<0.001; Table 3). Specific factors and compounds contributed differently to the observed chemical differences. Four out of the six factors and only ten compounds in these four chemical factors varied significantly between populations (Table 3).

**Table 3:** Multivariate and univariate analyses in chemical factors and chemical compounds between populations of *S. lamella*.

Chemical factors	F	P	Chemical compounds	F	P
**FC1**	**4.243**	**0.002**		**16.961**	**<0.001**
			18	Ns	
			19	Ns	
			17	Ns	
			23	7.269	0.010
			20	15.748	<0.001
			21	16.654	<0.001
			14	Ns	
FC2	Ns				
**FC3**	**2.947**	**0.014**		**44.223**	**<0.001**
			5	8.605	0.006
			4	4.295	0.045
			9	13.646	0.001
			7	30.78	<0.001
			(1) nitenin	172.782	<0.001
**FC4**	**2.775**	**0.020**		**6.267**	**0.002**
			2	Ns	
			3	18.611	<0.001
			8	Ns	
**FC5**	**21.830**	**<0.001**		**13.500**	**<0.001**
			6	12.892	0.001
			21	16.654	<0.001
FC6	Ns				

MANOVAs were run independently for each chemical factor. F-ratio and p-value of significant differences (P≤0.05) included. Ns: non-significant values.

**Figure 4:**
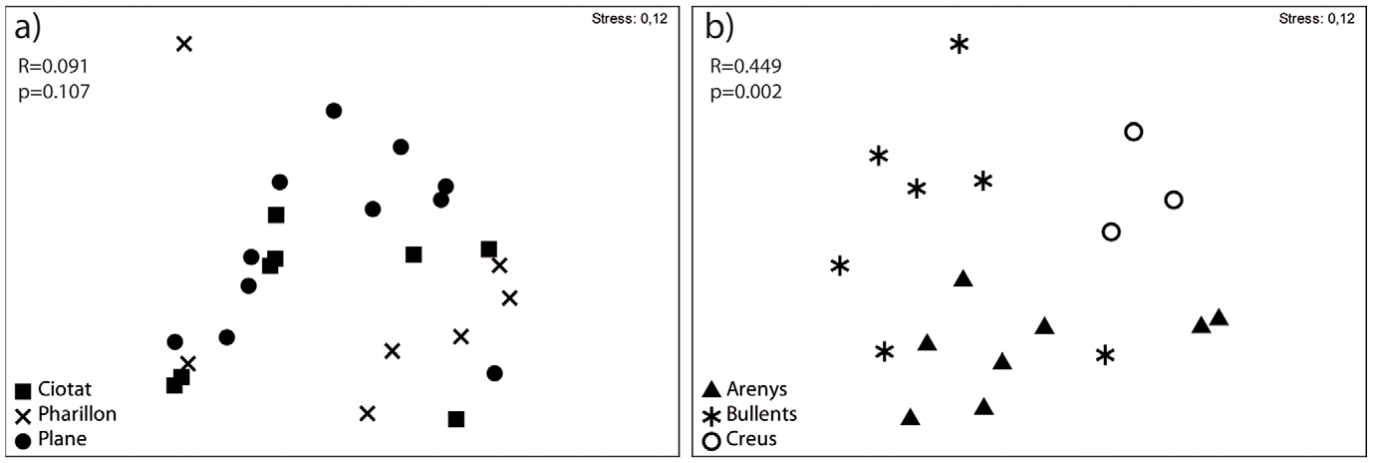
Chemical dissimilarities of *Spongia lamella*. Chemical dissimilarities were estimated by non-metric multidimensional scaling (MSD) and analysis of similarity (ANOSIM) from each the individual chromatograms using the 10 compounds responsible for variations, at local scale of Marseille a), and regional scale of Catalonia b).

**Figure 5:**
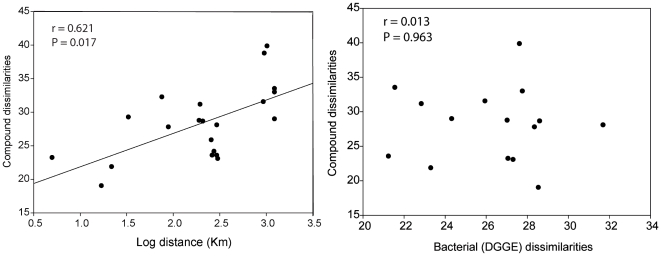
Relationship between chemical dissimilarities, geographical distances and bacterial dissimilarities. Chemical dissimilarities were estimated on the ten compounds responsible for variation between pair of sponge populations. Geographical distances were estimated as the logarithm of the geographical distances that separated pair of populations. Bacterial dissimilarities were estimated on the abundance/intensity of bacterial bands from DGGE gels using 4 sponge specimens per population. Chemical and bacterial dissimilarities values are included in table 4.

We did not find significant chemical differences in the ten variable compounds between local population of *Spongia lamella* (Plane, Pharillon and La Ciotat, distant by less than 25 km, one way ANOSIM, global R = 0.091, p = 0.107, Figure 4a); However at larger geographical scale (Catalonia), we found significant chemical differences between populations (Cap de Creus, Els Bullents and Arenys distant by over 100 km, one way ANOSIM, global R = 0.449, p = 0.002, Figure 4b). We also detected a significant correlation between the chemical dissimilarities obtained with the ten variable compounds (Table 4) and the geographic distances between populations (*Mantel test* P = 0.017, Figure 5a). Geographic distance explains 38.5% of the variance in chemical dissimilarity between sponge populations. Although we also found a relationship between bacterial dissimilarities and geographical distances between sponge populations [38], we failed to detect a correlation between chemical and bacterial dissimilarities (*Mantel test* P = 0.963, Figure 5b).

**Table 4:** Chemical (below the diagonal) and bacterial (above the diagonal) dissimilarities as estimated by the SIMPER procedure (PRIMER software) between pairwise sponge populations.

Area	Populations	Cio	Pla	Far	Cre	Bul	Are	Ceu
Marseille	Cio	-	28.55	23.31	28.61	-	27.32	27.77
	Pla	19.01	-	27.07	22.85	-	21.25	21.56
	Far	21.85	23.21	-	27.03	-	31.70	24.33
Catalonia	Cre	28.64	31.14	28.75	-	-	28.36	27.63
	Bul	24.14	22.56	25.84	32.24	-	-	-
	Are	23.06	23.54	28.07	27.77	29.25	-	25.95
Gibraltar	Ceu	32.97	33.50	28.97	39.84	38.77	31.53	-

Chemical dissimilarities were calculated from the 10 compounds that showed significant variations (Table 2). Bacterial dissimilarities were calculated from DGGE profiles of 4 specimens per populations (except for Els Bullents).

## Discussion

Understanding the intra-specific level of diversity in secondary metabolites is critical to advancing in chemical ecology [8]. However, studies addressing chemical diversity are underrepresented in marine systems [5], [8]. Within-species diversity provides the variance on which evolution acts [4], [5] and has important implications in population dynamics, conservation, and biotechnology [5], [42], [43], [44]. To our knowledge, and beyond the variation in secondary metabolism across locations, our study is the first report of a geographic pattern in chemical diversity at an intra-specific level. We have documented the chemical diversity within the sponge *Spongia lamella* from nearby locations a few kilometers apart to populations spread over 1200 km. Chemical diversity in this species was relatively low, given the nature of the chemical profiles: the presence of the 2 major compounds led to the uneven repartition of diversity. We found significant differences in both Shannon and Margalef's indexes at the population level, sponge populations varied significantly in chemical richness and diversity. However, no differences in these diversity indexes were noticed at a regional geographic scale. This supported the capital role of the population level in analyses of diversity, especially when dealing with multiple components of biodiversity [4]. We also found that chemical dissimilarity in *Spongia lamella* increased with increasing geographical distances between populations.

We know little about geographic patterns of secondary metabolites at an intra-specific level in marine organisms. Data on this particular area are sparse, with contrasting results, and sometimes with methodological constrains. A study on bromotyrosine derivatives from *Aplysina fulva* evidenced chemical variability among specimens collected in multiple locations in Brazil and in a South Atlantic Bight reef in the United States); but multiple extraction and isolation protocols hampered ecological conclusions [48]. In contrast, the occurrence of brominated compounds in specimens of the sponge *Aplysina aerophoba* collected around the Canary Islands were identical, although their concentration varied [26]. Variation in the occurrence of secondary metabolites as a function of geographic location occurs in species from multiple animal and vegetal phyla [27], [49], [50], [51]. The only geographic pattern in the production of secondary metabolites that has received attention is the latitudinal gradient hypothesis [52]. Yet, most evidence for or against this hypothesis stems from the activities of secondary metabolites rather than from secondary metabolism itself [53], [54], [55]. Within-species variation of secondary metabolites from multiple locations can be so high as to cast doubt on the role of latitude [56]. Our study proved that pattern detection is possible despite significant chemical variation.

Chemical profiles are an excellent approach to quantify the relative abundance of secondary metabolites and assess their variations [57], and to address three out of the four concepts on which diversity is based. The universal ELSD detector was found to be the most appropriate detection mode for the visualization of the global secondary metabolism of a desalted marine extract. We used UV and MS to confirm the identity of the main quantified compound but these chromatograms were less representative of the real chemical composition of our fraction than our ELSD profile. Indeed responses in UV or MS of some of the compounds could strongly differ depending on their structure. However, the use of ELSD detector as well as the 280 nm chromatograms under our HPLC conditions allowed the visualization of the second major secondary metabolite which had remained undetected so far. Our study on the chemical diversity of *S. lamella* led us to identify five known terpenes. An ergosterol derivative is for the first time described for this species [47] and maybe a precursor of the previously described oxidized ergosterols in this species [33]. Ergosterols and their ester derivatives have already been reported from several fungi and are known to exhibit physiological roles, ergosteryl esters being a supply storage form [58]. The ergosterol ester identified in *S. lamella* (compound **6**) was included in the Factor 2 (FC2) of the Factor Analysis which did not show significant variation between sponge populations. However, the role of this compound in the primary or secondary metabolism is not known. Previous studies reported that nitenin and dihydronitenin are the major compounds of *S. lamella* [31], [33], [59]. In agreement with these studies, we found nitenin in high abundance but we failed to detect a high amount of dihydronitenin. Dihydronitenin was identified by HPLC-MS, however its low concentration prevented any quantification.

Given the distance covered in our surveys (1200 km), factors such as water temperature, light exposure, food availability, and others varied tremendously between locations and could affect the production of secondary metabolites in this sponge [60]. A particular constraint we faced was the difficulty to find *S. lamella* in some locations. For example, we observed and collected a single sponge in Ceuta, which limits ecological interpretations. However, given its important geographic location, we believe it was worth considering the information that this single specimen provides, so we used it for our diversity and dissimilarity analyses. Nevertheless, the use of replicates in the remaining locations in Catalonia and Marseille makes analyses robust. Populations were collected as we became aware of their existence, which provides additional limitations to our data set. Because of a few samples collected in two seasons in Catalonia and Marseille, we could check for the effect of season in the variations of chemical profiles in our samples. With our data, seasonal differences failed to increase chemical dissimilarities between populations in Catalonia or Marseille, supporting the role of space rather than time in the chemical differences quantified in our study. However, seasonal variation in the production of secondary metabolites could occur in *S. lamella*, as it has been reported for other sponges [16], [61], [62], [63]. Moreover, temporal variation can be site specific [18] and could act as a confounding factor for spatial analysis even if samples are collected within the same season. Ideally, pluriannual studies at multiple locations are crucial to understand chemical diversity at an intra-specific level. Qualitative and quantitative differences in the metabolites of *Spongia lamella* occurred between populations. The pattern of increasing chemical dissimilarity with geographic distance between sponge populations was remarkable. Geographic distance explained nearly 40% of the variance in chemical dissimilarity.

Whether variation in secondary metabolites is genetic or environmentally controlled is still an unresolved issue. Spatial and temporal variations could affect trophic and competitive interactions, increasing the chemical patchiness of the environment and promoting biodiversity at both genetic and species levels [41]. Increasing small-scale heterogeneity is crucial to create opportunities for new species to settle down. Alternatively, sponge genetic diversity could promote chemical diversity if secondary metabolites are produced by the sponge and the production has a heritable basis. Production of a cocktail of chemical compounds could confer advantages to an individual sponge and promote survival when facing specific predators, competitors, pathogens or environmental factors. If so, chemical diversity would increase sponge fitness. However, if just a limited number of chemical compounds are advantageous, increasing diversity of chemical compounds with ecologically irrelevant metabolites might dilute this benefit.

Distinct patterns of variation in secondary metabolites could also result from the dynamic nature of true sponge cells and microbial communities. There is little doubt that sponge associated microbes could account for many of the secondary metabolites assigned to the host [64], so changes in microbial populations that produce secondary metabolites will certainly lead to changes in sponge secondary chemistry. In fact, there is considerable debate as to whether sponges, their associated endobionts, or both parts are responsible for the production of secondary metabolites [65]. *Spongia lamella* hosts a very abundant and dynamic bacterial community in the western Mediterranean [38], [66]. Our preliminary analyses showed independence between bacterial and chemical diversities [38]. Regardless bacteria do not influence or are influenced by metabolite production; secondary metabolites could protect sponges from pathogens by preventing them to overrun the sponge tissues. This would still have positive effects both on the sponge and associated bacterial communities, as it could limit competition between pathogens and symbiotic bacteria. Further analyses of these types of links between chemistry and bacteria would provide alternative ways to understand the production of secondary metabolites [67]. Clearly, this is a challenging area in sponge chemical ecology that will benefit tremendously from the new generation of genetic techniques [64].

Our study also highlights the complexity of the secondary metabolism within a single species. In *Spongia lamella*, we examined and quantified the relative abundance of 25 compounds that formed six coherent chemical factors. Ten compounds (41%) in four chemical factors varied significantly between populations while the remaining compounds showed no significant variation. Although uncertain at this time, these groups of compounds might represent inducible and constitutive secondary metabolites, although we cannot completely rule out the presence of some primary metabolites, particularly in the highly lipophilic section of the chemical profile. Constitutive defenses are always present in the organisms while inducible defenses can be synthesized or activated when needed [68], [69]. Although both types of defenses can vary spatially, inducible defenses have the additional benefit of increased variability [70]. In our samples, the major compound (nitenin) varied significantly between populations while the second major compound (ergosteryl myristate) showed no significant variation. This could suggest distinct roles of diverse metabolites, and highlighted the importance of secondary metabolites as response of populations to their environment. Some of the metabolites isolated from this sponge showed cytotoxic activities against multiple tumor cell lines [33] but the ecological roles remain mainly unknown. We need further research to assess the functional role of these compounds in nature and how they respond to biotic or abiotic variables. This would unravel the last aspect of biodiversity: the functional role, which could give a complete insight of the chemical diversity of this endangered sponge [45], [71].

### Conclusion

Beyond an exhaustive structural characterization of all compounds, our study assessed chemical diversity by looking relatively at the number of compounds, their abundances, and dissimilarities in multiple populations of *S. lamella*. Our data showed qualitative and quantitative variation in the chemical profiles between populations and distance appeared as a major mechanism of chemical differentiation in *S lamella*. Environmental and genetic factors could be responsible for such variation [72], [73]. In marine seaweeds, quantitative variation in furanones from *Delisea pulchra* showed significant heritability, indicating a potential for an evolutionary response [73], [74]. In fact, variability per se could be target of selection [70] and chemical diversity could promote species evolution and biodiversity through the variety of biotic interactions that it mediates [6], [41]. A better understanding of the patterns of chemical diversity is crucial to assess the mechanisms that generate and maintain chemical diversity and its consequences in species biology, ecology, and evolution.

## Materials and Methods

### Target Species

*Spongia agaricina* first description was performed on a now missing specimen from the Indian Sea [75]. Sharing similar morphology and shape with the Mediterranean sponge, the name *S. agaricina* was generalized and used for the Mediterranean specimens. Nowadays *S. agaricina* may be better known as a Mediterranean than Indian species. However, new evidence showed significant differences between the Philippine and Mediterranean specimens [75], [76], which should be referred to as *S. agaricina* Pallas 1766 and *Spongia lamella* Schultze 1879 respectively [75].

### Collection Sites

**Figure 6:**
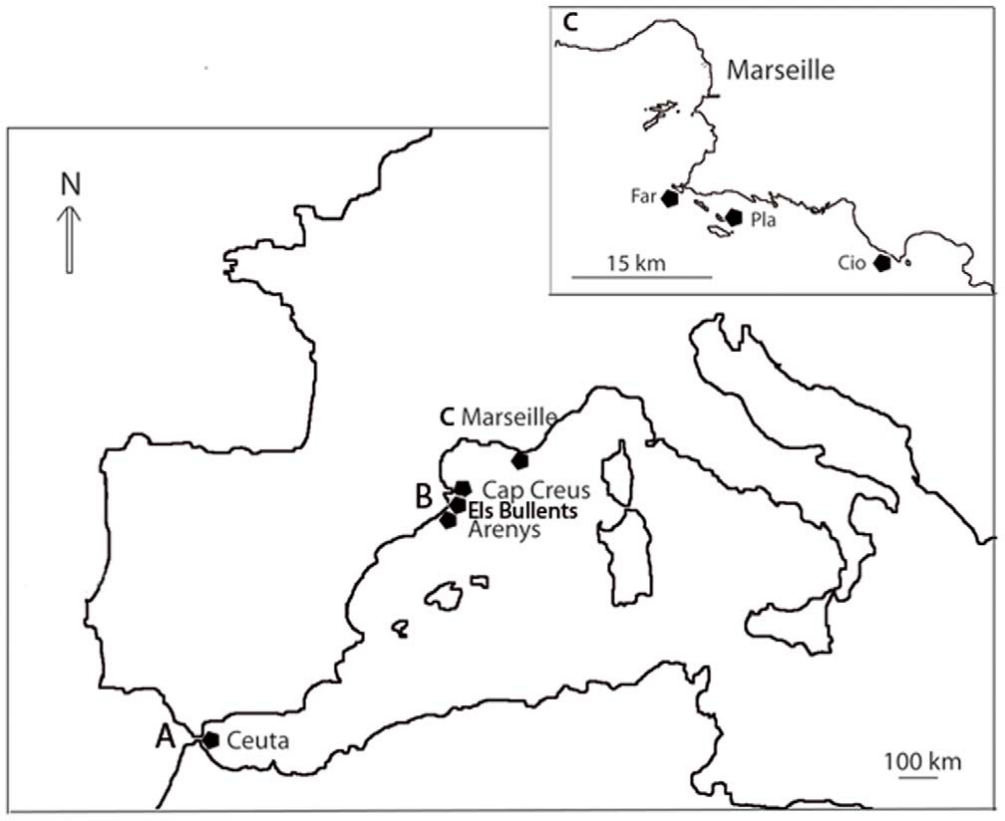
Map of sampling locations in the Western Mediterranean Sea. Samples were collected at various geographical scales in Ceuta (A), Catalonia (B) and in Marseille (C) areas. The area of Catalonia (regional scale) included populations at Cap Creus, Els Bullents, and Arenys; and the area of Marseille (local scale) included populations at Plane (Pla), Pharillon (Far) and La Ciotat (Cio).

We sampled a total of 44 specimens from six populations of *Spongia lamella* in two geographic areas from the Northwestern Mediterranean Sea: Marseille (France), Catalonia (Spain), and an additional specimen from Ceuta (Strait of Gibraltar) (Figure 6). In December 2006, we collected 24 specimens by SCUBA diving in three locations near Marseille that spread over a distance of 22 km (Pharillon: 43°12′N, 5°20′E; Plane: 43°11′N, 5°23′E; La Ciotat: 43°9′N, 5°35′E). We also collected three additional samples around Plane in July 2007 and June 2008. In Spain, 18 more specimens were collected by SCUBA from three locations spread over a distance of 100 km (Arenys de Mar: 41°34′N, 2°33′E; Els Bullents: 41°42′N, 2°53′E; Cape Creus: 42°17′N, 3°18′E). Samples were collected as we became aware of populations with multiple specimens in November 2006 (Arenys de Mar), June 2006 (Cap de Creus), and Els Bullents (July 2008). In June 2007, a single specimen was observed and collected in Ceuta (35°53′N, 5°17′O).

All samples were individually placed underwater in plastic bags containing seawater, placed in a cooler for about 1 hr to reach the shore, and then manually cleaned to remove epibionts. They were then placed back in their plastic bags in the cooler to be transferred to the laboratory where they were placed in a freezer at −20°C. All specimens were then freeze-dried and stored in a −20°C freezer until chemical extraction.

### Sample Preparation and Chemical Profiles

This analytical process has already been used for a chemotaxonomic approach among Homoscleromorpha sponges [77]. A piece of the sponge was freeze-dried and ground to yield a fine and homogenous powder. 20 ml of a 1∶1 volumetric mixture of MeOH/CH_2_Cl_2_ were then added to a beaker containing two grams of this powder (dry mass). After 10 min in the ultrasonic bath, the organic phase was carefully recovered by pipeting and the process was repeated twice. The three organic phases were pooled and evaporated under reduced pressure after addition of 1 g of C_18_ silica powder. Desalting was then performed by SPE on a Phenomenex Stata™ C_18_ cartridge previously washed with 10 ml of MeOH/CH_2_Cl_2_ 1∶1 and conditioned with 10 ml of water. The cartridge containing the solid extract was first washed with 50 ml of milliQ to remove salts elution with MeOH/CH_2_Cl_2_ 1∶1 yielded the organic phase. Ten µl of this organic fraction were then analyzed by HPLC composed of a Waters™ 717plus autosampler, a Waters 600 controller, a Waters 996 photodiode array detector (DAD), and a SEDERE France evaporative light scattering detector (ELSD) to obtain quantitative profiles (Figure 3). High performance liquid chromatography (HPLC) experiments were run on a Phenomenex Gemini C_6_-Phenyl (250×3 mm, 5 µm) column, using a linear gradient elution of A (H_2_O+0.1% formic acid v/v) and B (acetonitrile+0.1% formic acid v/v) from 90% A at t 5 min to 100% A at t 35 min then isocratic until t 45 min (flow 0.5 mL.min^−1^). Ultraviolet (UV) chromatograms were extracted at 210, 254, and 280 nm from the DAD detector. ELSD profiles were also used for chemical fingerprints. An Esquire 3000 Bruker mass spectrometer (MS) was also used with this HPLC system for *m/z* determination of selected compounds. HPLC-MS were run on a single randomly selected sample for each population. Data were visualized and processed by Bruker Daltonics Data Analysis 3.4.

Chromatographic profiles of all samples were processed by the Empower software with automatic integration of peak area on ELSD profiles. Integrations were individually checked to remove any artifacts. Nitenin was used as an internal standard. After background subtraction, the relative abundance of each compound was measured by integrating the area under the peak and was expressed as percentage of the total abundance in the chromatogram. Relative peak area allows an accurate and relative quantification of peaks between sponge samples. We calculated the absolute abundance of each compound proportionally to the nitenin concentration.

### Isolation of Nitenin and Quantification

Three sponge samples from populations of Marseille were used for secondary metabolites isolation and identification. A pool of 25 g of sponges (dry mass) was extracted as described before, deposited on 2.5 g of C_18_ phase and subjected to Flash Chromatography on a C_18_ silica column (Armen Spot Flash, 10 mL.min^−1^). The crude extract was fractionated with 250 mL of 1) H_2_O 100%, 2) H_2_O/MeOH 1∶1, 3) MeOH 100%, 4) MeOH/CH_2_Cl_2_ 3∶1, and 5) in CH_2_Cl_2_ 100%. HPLC-MS and nuclear magnetic resonance (NMR) analyses of the fractions showed that nitenin was a pure compound in fraction 3 (564 mg) [30], and was also present as a mixture in other fractions. A calibration curve of nitenin was obtained by HPLC-ELSD using seven MeOH/CH_2_Cl_2_ 1∶1 solutions of nitenin at concentrations ranging from 0.08 mg to 10 mg/ml. Nitenin concentration in each sample was quantified by relating the peak area in the ELSD profile to the standard curve.

### Isolation of the major lipophilic compound

For isolation of the second major compound, HPLC was conducted on the same equipment as previously described. The sample extract 4 (MeOH/CH_2_Cl_2_ 3∶1) was separated and analyzed by using a Semi-preparative Diol Hypersil column in a normal phase at room temperature. We used an isocratic gradient consisting of solvent A (Ethyl acetate, 12%) and solvent B (hexane, 88%). Chromatographic peaks were identified by comparing the retention times, and UV spectra. After peak recollection, NMR spectra were recorded on a Bruker Avance 500 spectrometer in CDCl3 (Figure S1).

### Bacterial profiles

We used the universal bacterial primers BAC358F (5′- CCT ACG GGA GGC AGC AG -3′) and BAC907RM (5′- CCG TCA ATT CMT TTG AGT TT -3′) to amplify fragments approximately 560 bp long in 4 specimens per sponge populations (except for Els Bullents population sampled afterward). We then performed Denaturing Gel Gradient Electrophoresis (DGGE) to obtain a first insight into the bacterial diversity inhabiting the sponges. DGGE analysis was performed using a Bio-Rad Dcode universal Mutation Detection System (BioRad) on 6% polyacrylamide gel in 1× TAE (40 mM Tris Base, 20 mM Sodium acetate trihydrate and 1 mM EDTA). We used a 40–75% vertical denaturant gradient (100% denaturant agent is 7 M urea and 40% deionized formamide). Comparable amounts of PCR products were loaded for each sample. Gels were run for 5 hours at 195 V at 60°C. Images of the gels were analyzed using the Gels plot lanes tool of ImageJ software 1.38× (Wayne Rasband, National Institutes of Health, USA). After background subtraction, the intensity of each band was measured by integrating the area under the peak and was expressed as percentage of the total intensity in the lane [38], [66]. We measured the relative abundance of each band and compared bacterial profiles among and within sponge populations. We considered bacterial richness as the number of bacterial bands, and evenness as relative intensity of these bands [67].

### Data Analysis

We tested for differences in chemical diversity between populations and geographical regions. For each specimen, we quantified the compound richness (Species Richness Margalef's index) and the Shannon's index using the DIVERSE procedure available in PRIMER 6 (Clarke and Warwick, 2001 Plymouth Marine Laboratory, UK [78]). Margalef's index is widely used in ecological studies to provide a measure of species richness (SR). SR is roughly normalized for sample size and is calculated as SR = (S−1)/log (N), where *N* is the number of individuals per population and S is the number of species. Using this index in our data, N was the abundance of sponge chemical compounds in a population and S the number of chemical compounds (e.g. number of peaks on the profiles). Chemical diversity was also calculated using Shannon's index, *H* (*H* = -**∑** *pi* log(*pi*), where pi is the proportion i.e. abundance of the ith compound of an individual chromatogram). We used SYSTAT 12 software to perform analyses of variance (one-way *ANOVA*) on both SR and H.

Given the large number of secondary metabolites analyzed in this study, we used factor analysis (FA) to look for independent chemical factors formed by related chemical compounds [67], [79]. Specifically, we used a principal component analysis extraction (PCA) with varimax rotation. The varimax rotation minimizes the number of variables that load highly on a factor and maximizes the loading variance across factors, which facilitate factor interpretation. The resulting independent factors were used as chemical variables in our analyses.

Because samples were collected over several seasons, we first tested whether seasonality could distort our spatial analysis. To do so, we used t-tests to compare the extent to which chemical dissimilarities within and between seasons differed in Marseille and Catalonia. We used the Multivariate Analysis of Variance (*MANOVA*) procedure in SYSTAT 12 to test whether chemical factors differed between sponge populations and to identify the secondary metabolites behind any significant factor.

We also performed statistical calculations available in the PRIMER v6.1.9 computer program [75] to analyze differences in the 10 compounds as function of sponge population. Square root data were used to calculate Bray-Curtis similarity. Chemical dissimilarities were analyzed graphically non-metric multidimensional scaling (MDS), and statistically by Analysis of Similarity (ANOSIM) at local (Marseille) and regional (Catalonia) scale. An exploratory similarity breakdown using the SIMPER procedure was used to the chemical dissimilarities between pair of sponge populations (percentages of dissimilarities between groups of samples). We then used a Mantel test to investigate whether 1) chemical dissimilarity and geographical distance, and 2) chemical and bacterial dissimilarities were related. Since the Mantel test can detect a significant association between two matrices we built a distance matrix with the geographical distances between populations, and matrixes containing the chemical dissimilarities, and bacterial dissimilarities between populations, respectively, as obtained from the SIMPER procedure.

## Supporting Information

Figure S1 **RMN spectrum of ergosteryl myristate −1[H+] in CDCl3.**
(PDF)
