# Supplementary figures and images for: Patterns of Chemical Diversity in the Mediterranean Sponge Spongia lamella
Source: PLoS One. 2011 Jun 17;6(6):e20844. doi: 10.1371/journal.pone.0020844 (PMC3117848; doi:10.1371/journal.pone.0020844)

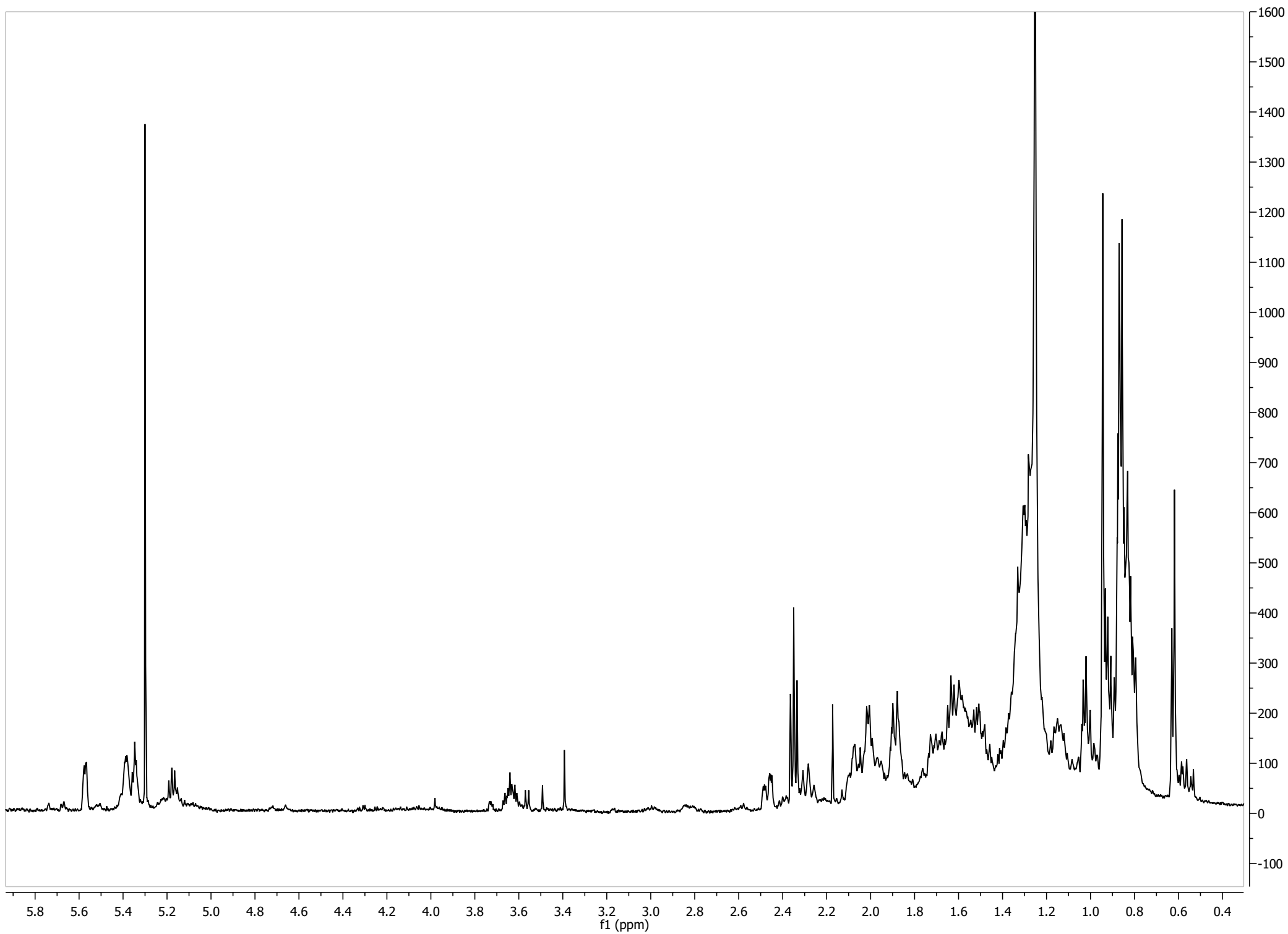

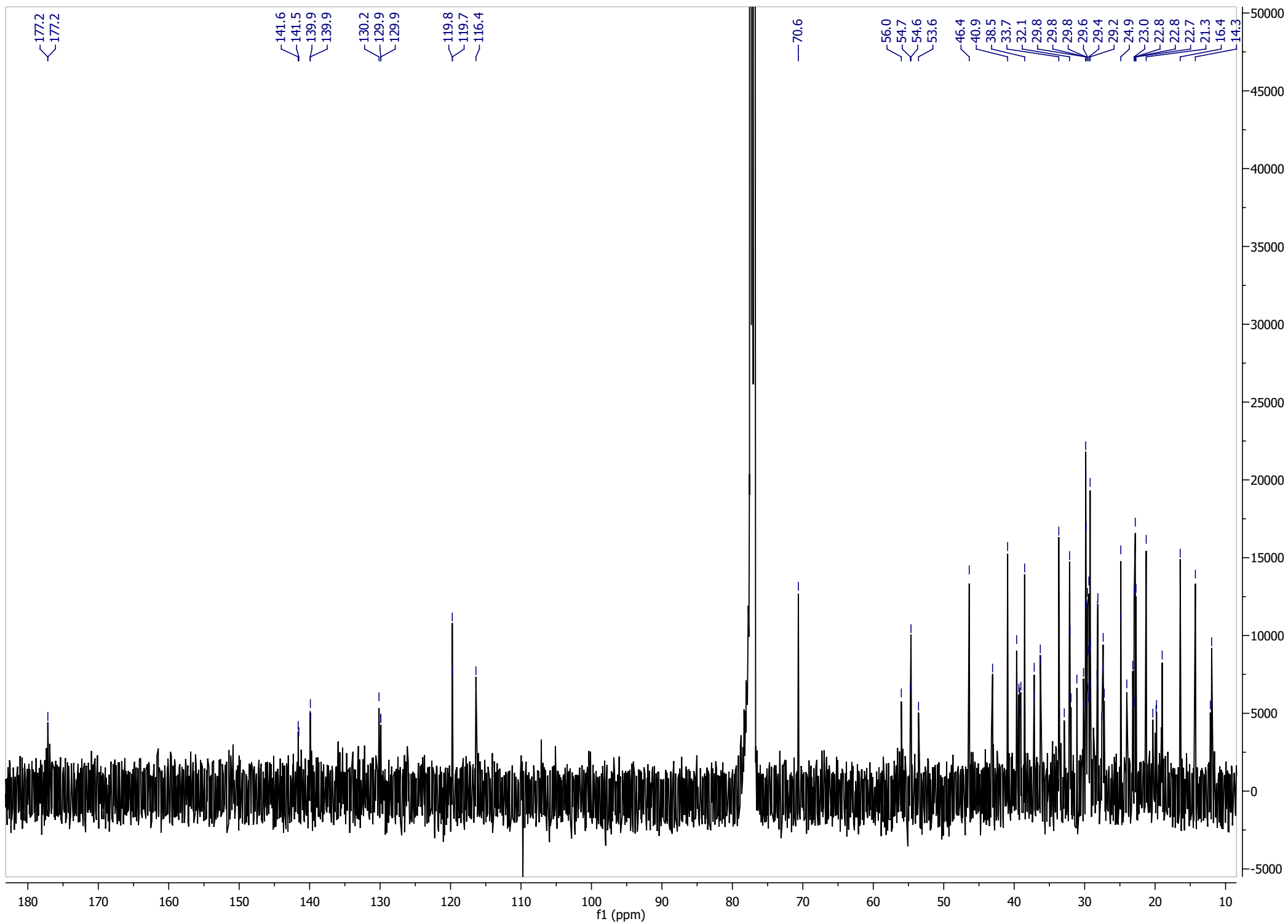

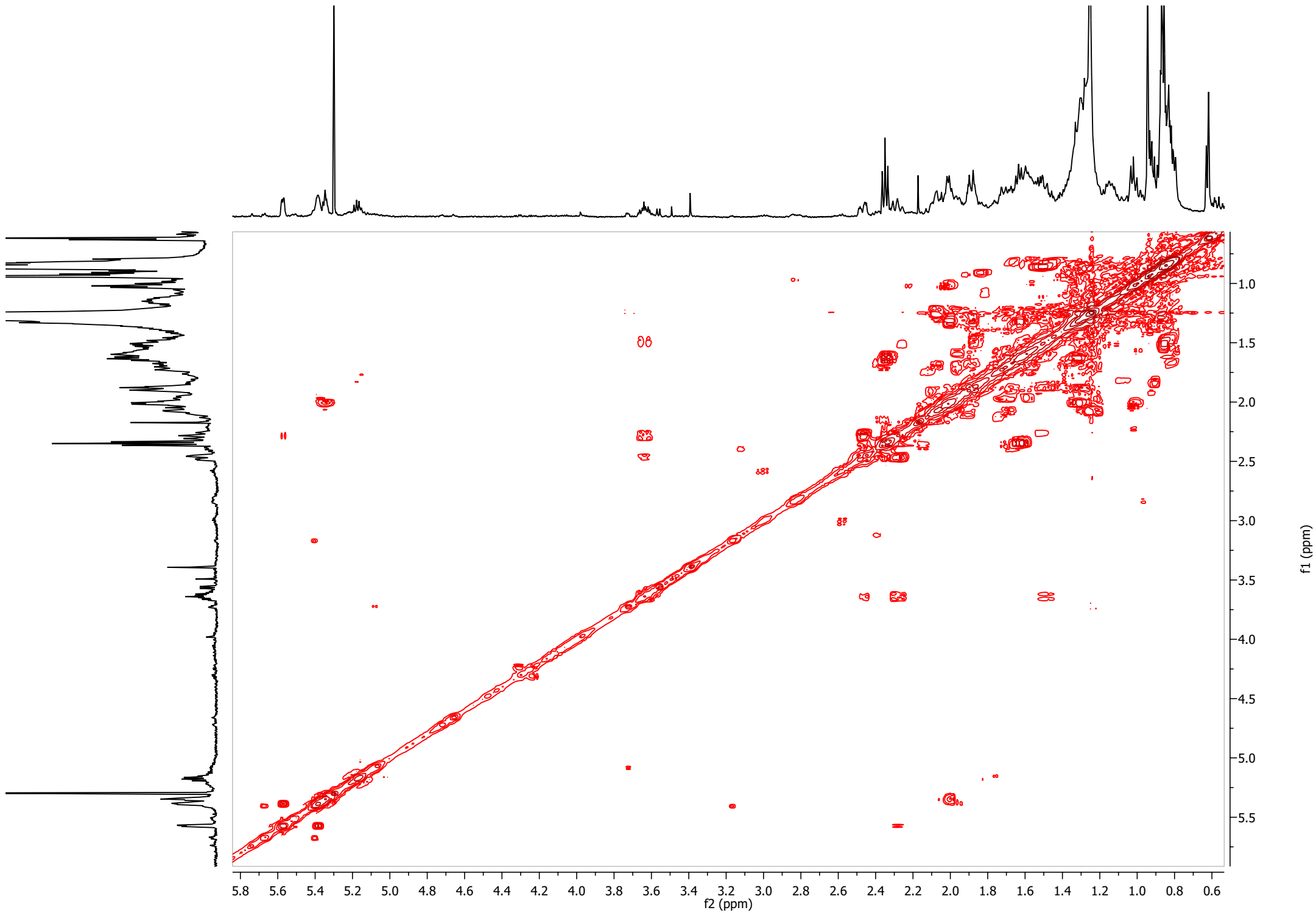

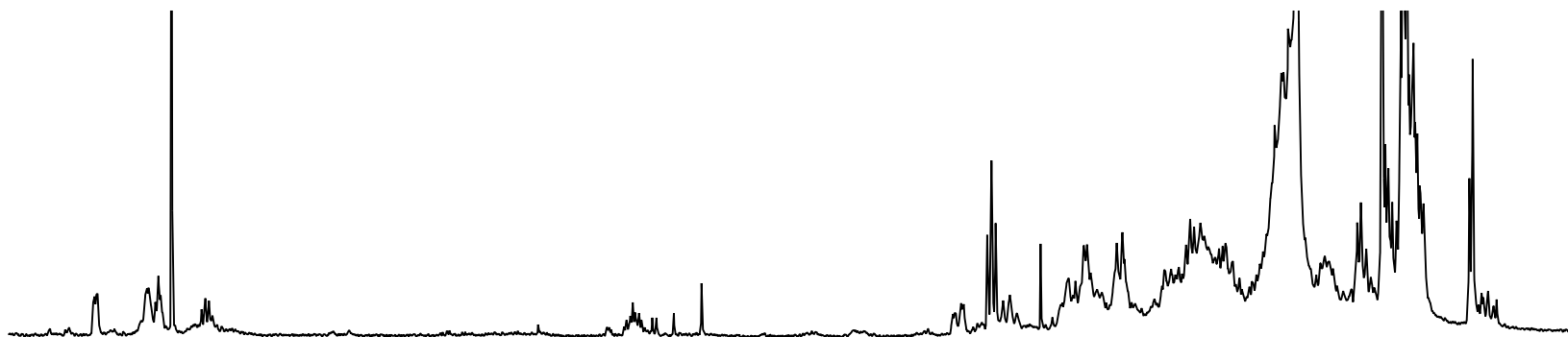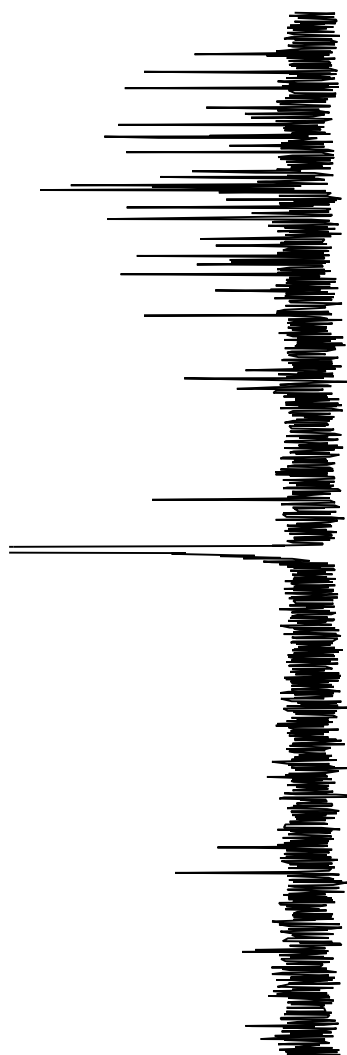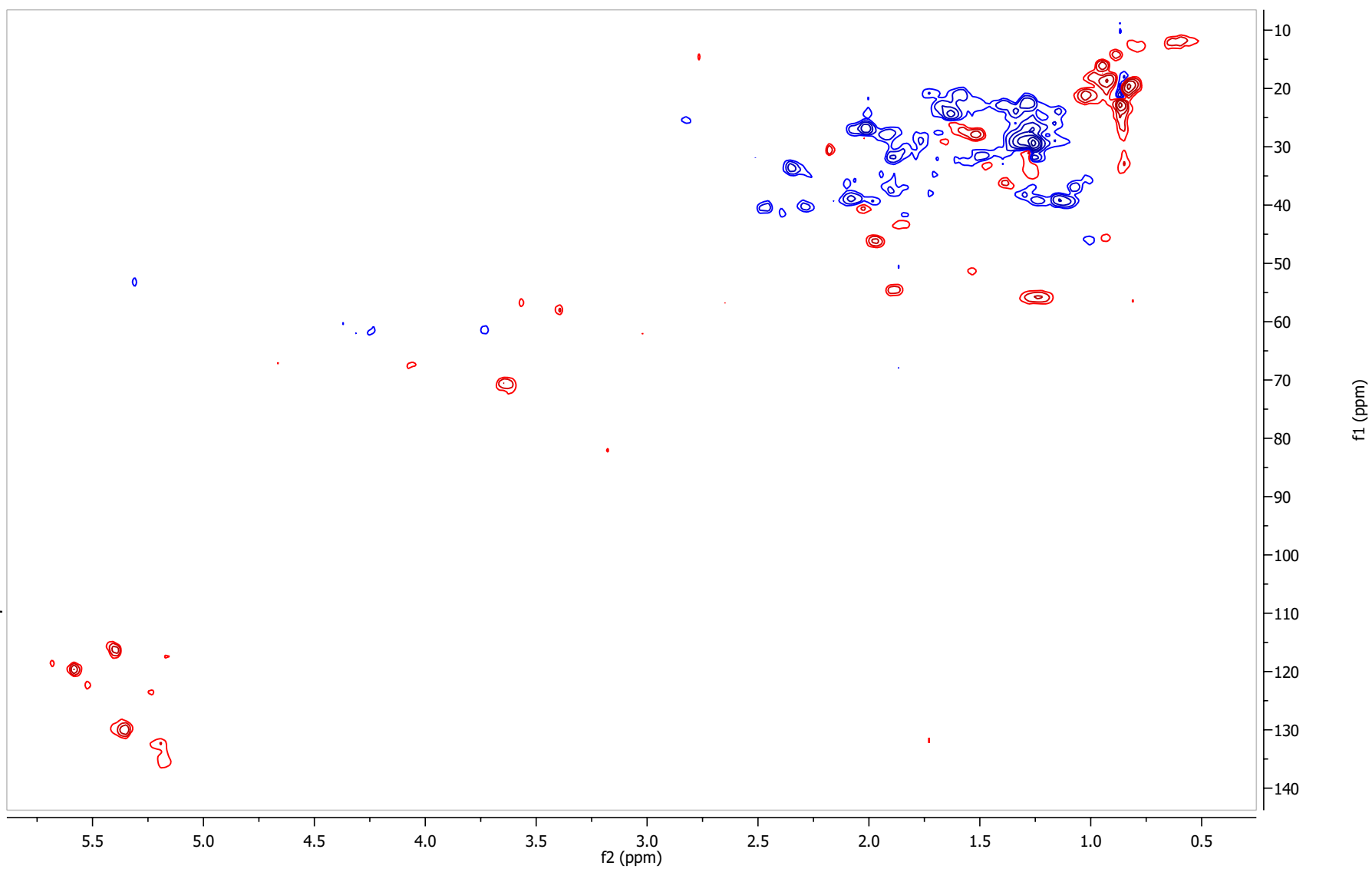

Supplement: Figure S1 — RMN spectrum of ergosteryl myristate −1[H+] in CDCl3. (PDF) [file pone.0020844.s001.pdf]
